# Implementation of deep reinforcement learning models for emotion detection and personalization of learning in hybrid educational environments

**DOI:** 10.3389/frai.2024.1458230

**Published:** 2024-11-28

**Authors:** Jaime Govea, Alexandra Maldonado Navarro, Santiago Sánchez-Viteri, William Villegas-Ch

**Affiliations:** ^1^Escuela de Ingeniería en Ciberseguridad, FICA, Universidad de Las Américas, Quito, Ecuador; ^2^Maestría en Seguridad Digital, Escuela de Postgrados, Universidad de Las Américas, Quito, Ecuador; ^3^Departamento de Sistemas, Universidad Internacional del Ecuador, Quito, Ecuador

**Keywords:** artificial intelligence in education, deep reinforcement learning, emotion detection, personalization of learning, computer vision

## Abstract

The integration of artificial intelligence in education has shown great potential to improve student’s learning experience through emotion detection and the personalization of learning. Many educational settings lack adequate mechanisms to dynamically adapt to students’ emotions, which can negatively impact their academic performance and engagement. This study addresses this problem by implementing a deep reinforcement learning model to detect emotions in real-time and personalize teaching strategies in a hybrid educational environment. Using data from 500 students, captured through cameras, microphones, and biometric sensors and pre-processed with advanced techniques such as histogram equalization and noise reduction, the deep reinforcement learning model was trained and validated to improve the detection accuracy of emotions and the personalization of learning. The results showed a significant improvement in the accuracy of emotion detection, going from 72.4% before the implementation of the system to 89.3% after. Real-time adaptability also increased from 68.5 to 87.6%, while learning personalization rose from 70.2 to 90.1%. K-fold cross-validation with k = 10 confirmed the robustness and generalization of the model, with consistently high scores in all evaluated metrics. This study demonstrates that integrating reinforcement learning models for emotion detection and learning personalization can transform education, providing a more adaptive and student-centered learning experience. These findings identify the potential of these technologies to improve academic performance and student engagement, offering a solid foundation for future research and implementation.

## 1 Introduction

In recent years, integrating artificial intelligence (AI) in education has shown significant potential to transform students’ learning experience. Among the most promising applications are emotion detection and learning personalization systems, which use advanced technologies to adapt to students’ emotional and academic needs in real time (Ouyang and Jiao, 2021). This study is part of this line of research, exploring the implementation of a deep reinforcement learning (DRL) model together with convolutional neural networks (CNN) and recurrent neural networks (RNN) to improve the accuracy of emotion detection and the personalization of learning in a hybrid educational environment (Zhang et al., 2023).

The motivation behind this research lies in the growing evidence that emotions play a crucial role in the learning process—previous studies, such as those by Trigueros et al. (2019), have shown that positive emotions can increase motivation and academic performance. In contrast, negative emotions can have the opposite effect. However, many existing studies focus solely on static data sets or predefined scenarios, limiting their ability to adapt to students’ evolving emotional states in real-time. Additionally, although CNNs and RNNs have been successfully applied in emotion detection tasks, their isolated use presents challenges in simultaneously handling spatial and temporal data. There is also a lack of research that integrates reinforcement learning models, like DRL, with emotion detection systems to optimize personalized learning strategies dynamically.

A critical gap in current research is the limited adaptability of existing models to diverse and fluctuating emotional and academic inputs in real-world educational environments. Most models cannot continuously adjust their strategies based on real-time emotional feedback, essential for effectively personalizing learning experiences. This gap highlights the need for more dynamic systems capable of handling complex emotional data and providing real-time personalization.

The main objective of this study is to design and implement an AI-based system that uses a hybrid model of CNN and RNN along with DRL to detect emotions in real-time and personalize teaching strategies based on these detections (Kotwal et al., 2022). The hypothesis is that such an adaptive system will improve accuracy in emotion detection and allow for more effective personalization of learning, ultimately improving academic performance and student engagement.

Emotional and academic data was collected from 500 students in a hybrid educational environment to address this goal. The data was captured using cameras, microphones, and biometric sensors and then pre-processed using advanced techniques such as histogram equalization and noise reduction to improve the quality of the inputs (Kumar and Mahajan, 2019). CNN was implemented for facial expression recognition and RNN for audio sequence analysis, combining both approaches in a hybrid model integrated with the DRL. The resulting model was trained using 70% of this data, with 15% allocated to validation and another 15% to testing.

By integrating CNN and RNN with DRL, this study addresses the gaps above by providing a system that processes spatial and temporal emotional data and dynamically adapts to students’ needs in real time. This hybrid approach allows for continuous personalization of learning strategies, which has been a significant limitation in previous research. Furthermore, using DRL enables the system to optimize decision-making processes, ensuring that the learning strategies evolve alongside students’ emotional and academic progress.

The study’s results are promising; the accuracy of emotion detection increased significantly from 72.4% before system implementation to 89.3% after implementation. Additionally, the system’s real-time adaptive capacity improved from 68.5 to 87.6%, and learning personalization increased from 70.2 to 90.1%. These results indicate that the system based on a hybrid model of CNN-RNN and DRL can detect emotions accurately and adapt teaching strategies effectively to meet students’ individual needs. K-fold cross-validation with k = 10 was used to evaluate the model’s robustness and generalizability. The results showed consistently high scores on all assessed metrics, suggesting that the model is stable and generalizable to different data set partitions. This robustness is crucial to ensuring the system can be applied effectively in various educational contexts.

The obtained results validate the effectiveness of combining CNN, RNN, and DRL for emotion detection and personalized learning. It fills critical gaps in research by providing a model that can dynamically adapt to students’ emotional states in real-time. These demonstrate the potential of advanced AI technologies to transform education, offering a more adaptive, responsive, and student-centered learning experience, which current models have not yet fully achieved.

This study significantly contributes to personalized education by integrating advanced AI models for emotion detection and learning personalization, specifically CNN, RNN, and DRL. The results validate the model’s effectiveness and highlight the potential of these technologies to transform education, providing a more adaptive and student-centered learning experience. This innovative approach can significantly affect how learning is approached in the future, improving student engagement and academic performance (Croll et al., 2023).

## 2 Literature review

Implementing intelligent systems for emotion detection and the personalization of learning in educational environments has gained increasing attention in recent research. This interdisciplinary field combines advances in AI, psychology, and educational sciences to improve students’ learning experiences by adapting teaching strategies to their emotional and academic needs (Coraci et al., 2023). Real-time emotion detection has become a critical tool for enhancing the quality of education, with studies highlighting how emotions impact students’ motivation, attention, and academic performance Villegas-Ch et al. (2023). These studies underscore the importance of systems capable of dynamically detecting and responding to students’ emotions to optimize their educational experience.

Despite these advancements, many current systems struggle to adapt to fluctuating emotional states in real-time, leading to limited personalization of learning strategies. A significant gap in the existing literature is the lack of integrated approaches simultaneously processing spatial (facial expressions) and temporal (emotional progression) data. Many systems employ either CNNs or RNNs in isolation. This restricts their ability to provide comprehensive emotion detection and learning personalization, particularly in a hybrid learning environment with constant emotional and academic variables.

Various approaches have been proposed for emotion detection, mainly using CNNs for facial expression recognition—for instance, Wang et al. (2023) demonstrated that CNNs could accurately recognize emotional expressions. However, the performance of such systems is often constrained by input data quality and the algorithms’ ability to handle variations in facial expressions. Our study addressed these challenges by applying advanced preprocessing techniques, such as histogram equalization and noise reduction, which significantly improved the quality of facial images used for model training, resulting in an emotion detection accuracy of 89.3%.

One of the primary limitations in the current literature is the reliance on static datasets and predefined scenarios for emotion detection, which fails to account for the dynamic nature of emotional states in real-world educational settings. This gap is particularly significant when attempting to personalize learning strategies, as current systems lack the flexibility to adjust in real time based on emotional feedback from students. This limitation demonstrates the need for more adaptive systems to continuously monitor and adjust teaching strategies to reflect students’ emotional and academic progress.

DRL methods have increasingly incorporated CNNs or RNNs to enhance their performance in various applications. CNNs are particularly effective in extracting spatial features from images, making them suitable for emotion detection from facial expressions. However, while efficiently handling spatial data, CNN-based methods face limitations when processing sequential information, such as emotional changes over time. On the other hand, RNNs, especially Long Short-Term Memory (LSTM) networks, are designed to handle temporal dependencies and are often employed for tasks requiring the analysis of time-series data. Despite their strengths in capturing temporal information, RNN-based methods struggle with spatial feature extraction and may suffer from vanishing gradient problems when processing long sequences.

However, the isolated use of CNNs or RNNs is insufficient to handle the complex emotional and academic inputs needed for effective real-time personalization of learning strategies. The literature lacks models that effectively combine spatial and temporal data processing with robust policy optimization, essential for adapting teaching strategies dynamically in response to students’ evolving emotional states.

Our approach combines the strengths of both CNNs and RNNs to manage spatial and temporal data more effectively. Our model can deliver a more comprehensive analysis by integrating CNNs for emotion recognition from facial features and RNNs for analyzing the temporal progression of these emotions. Furthermore, we employ Proximal Policy Optimization (PPO) to optimize this hybrid model in a dynamic educational environment. PPO has been shown to outperform traditional optimization methods by providing more stable and efficient learning, which is crucial when adapting teaching strategies based on students’ real-time emotional and academic feedback.

Research by Pervin et al. shows that the ability of a system to adapt in real-time to students’ needs is essential for personalized education—research by Pervin et al. (2021) tutoring systems can adapt educational content based on student interactions and performance. While CNNs are effective for emotion detection, the ability of a system to adjust teaching strategies in real time based on detected emotions is essential for personalized education. Deep reinforcement learning (DRL) has emerged as a powerful approach for real-time adaptation in educational environments (Petch et al., 2024) showed how DRL models could learn optimal policies for personalizing learning based on students’ emotional states and performance metrics. These models, however, face challenges in balancing exploration and exploitation in dynamic educational environments where emotional data is highly variable.

In DRL, two major categories of approaches exist: model-based and model-free. Model-based DRL methods, such as Dyna-Q, attempt to create a model of the environment, enabling simulations to predict future states and optimize decision-making (Lin et al., 2024). However, model-based methods may struggle to build accurate and timely representations of the environment in complex and unpredictable environments like education, where emotions fluctuate rapidly. On the other hand, model-free approaches, such as Q-learning and SARSA, directly optimize the agent’s actions without requiring an environmental model. Still, they are less effective in continuous or high-dimensional spaces, such as those needed for personalized learning based on emotions (Cloude et al., 2024).

PPO has gained recognition for its balance between stability and efficiency among the various DRL algorithms. PPO, a model-free method, has been particularly effective in tasks involving continuous action spaces, such as adjusting teaching strategies in real time to match students’ fluctuating emotional and academic states. Unlike algorithms like Deep Q-Network (DQN), which perform well in discrete action spaces but struggle with continuous outputs, PPO uses a clipped surrogate objective function that ensures stable learning without drastically altering the policy (Wang et al., 2023). This makes PPO a more suitable choice for our educational environment, where continuous adjustments to teaching strategies are needed based on subtle changes in student emotions.

While other algorithms like Advantage Actor-Critic (A3C) have been used in similar applications, PPO’s lower sensitivity to hyperparameters and better sample efficiency provided a more robust solution in our context. Previous studies employing Deep Deterministic Policy Gradient (DDPG) and A3C reported challenges with convergence in dynamic educational environments, where the state space continuously evolves due to students’ emotional and academic variability.

Our proposed method, which combines CNNs for spatial emotion recognition, RNNs for temporal analysis, and PPO for policy optimization, demonstrated superior adaptability and precision in real-time environments. This hybrid model significantly outperformed standalone CNN or RNN approaches, achieving an 88.0% precision rate compared to 85.6% for CNN-based and 83.2% for RNN-based methods. This improvement highlights the advantages of integrating spatial and temporal data processing with stable policy optimization, making our approach more effective for emotion-driven personalized learning in hybrid educational environments.

In this study, the PPO algorithm significantly improved real-time adaptability, increasing from 68.5 to 87.6%. This improvement can be attributed to the algorithm’s ability to maintain a stable learning process while effectively managing the variability of emotional data. Cross-validation techniques such as k-fold (k = 10) were applied to evaluate the model’s robustness, confirming consistently high scores in all performance metrics. Valuing intelligent educational systems is crucial to ensure their effectiveness and generalization. Studies such as those by Aldriwish (2024) have used cross-validation techniques to evaluate the robustness of these systems. K-fold cross-validation has effectively assessed the consistency of model performance across different partitions of the data set.

Some emerging reinforcement learning (RL) approaches have recently been proposed in model-based and model-free categories. These include Fujimoto et al. (2024), which aims to improve the representation of the state-action space, improve sampling efficiency, and simplify model-free RL architectures. Furthermore, Shang et al. (2023) and Huang et al. (2024) presents innovative techniques for regulating continuous actions in reinforcement learning.

While promising and offering potential advances in stability, efficiency, and adaptability within complex action spaces, our choice of PPO is supported by its demonstrated robustness in managing high variability and continuous adaptation, both essential in dynamic educational settings. Unlike Shang et al. (2023) and Huang et al. (2024), which is highly specialized for controlled environments, PPO has proven adaptable across fluctuating emotional and academic data in real-world applications. Future work may consider these approaches for specific scenarios once they have been validated through formal peer review. Still, for the current study, PPO aligns best with our goal of stable, continuous personalization in real-time educational contexts.

This study highlights the importance of selecting appropriate DRL algorithms for specific educational applications. It reinforces the suitability of PPO for real-time emotion detection and learning personalization in hybrid educational environments. The results provide a strong foundation for future research and further improvements.

## 3 Materials and methods

### 3.1 Description of the proposed system

This work proposes a system that uses DRL models to adapt real-time teaching strategies based on students’ emotions. Educational systems’ ability to recognize and respond to students’ emotions is crucial to fostering a positive and effective learning environment. By integrating advanced emotion detection technologies and DRL algorithms, this system seeks to personalize the educational experience, optimizing students’ academic performance and emotional well-being.

#### 3.1.1 General system architecture

The proposed system for emotional personalization in education using DRL comprises several integrated components that work together to detect students’ emotions and adapt teaching strategies in real-time. The system’s architecture is divided into three main modules: the emotional data capture module, the emotion processing and detection module, and the DRL-based educational adaptation and personalization module (Ducrocq and Farhi, 2023).

The emotional data capture module uses various devices to collect relevant information about the student’s emotional state. These devices include cameras to capture facial expressions, microphones for voice analysis, and biometric sensors to measure variables such as heart rate. The data captured by these devices is sent to the emotion processing and detection module, which is pre-processed and analyzed using AI techniques.

The emotion detection and processing module employs CNN and RNN to extract emotional features from facial images, voice signals, and biometric data. These characteristics classify the student’s emotional state in real time. The results of emotion detection are sent to the educational adaptation and personalization module. The educational adaptation and personalization module is based on a DRL model that dynamically uses the detected emotional information to adapt teaching strategies (Patel, 2023). This model comprises a learning agent that interacts with the educational environment, receiving inputs from the student’s emotional state and academic performance. DRL’s agent reward feature maximizes student engagement and performance by adjusting real-time educational activities and teaching methods.

**Figure 1:**
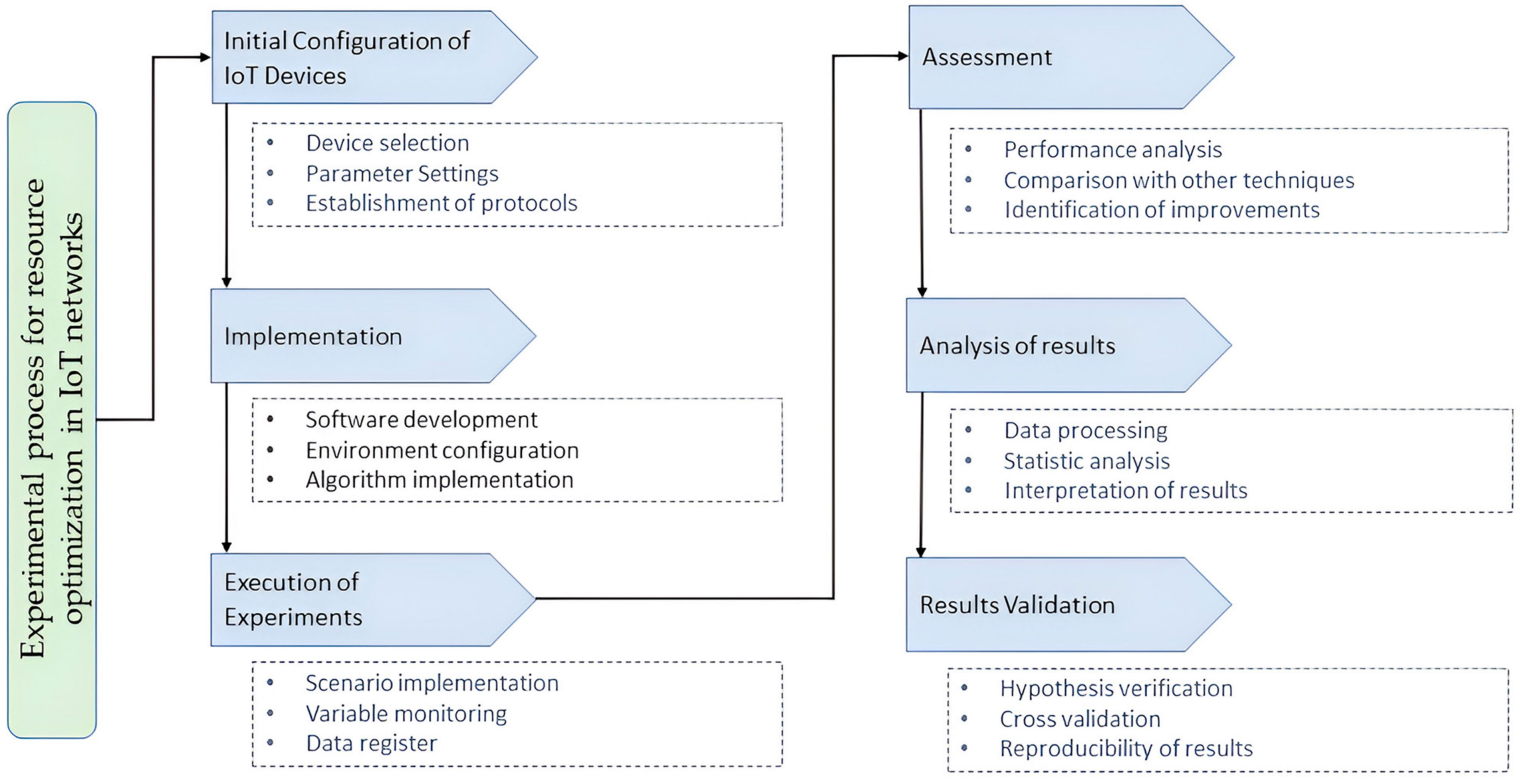
System architecture using deep reinforcement learning models.

The architecture is presented in the diagram in Figure 1, which shows the interaction between the different modules. The data flow begins with capturing emotional information, continues with emotion processing and detection, and culminates with the personalized adaptation of educational content using the DRL agent.

#### 3.1.2 Tools and technologies used

Implementing this system requires specific software, hardware tools, and advanced AI libraries and frameworks. Regarding hardware, high-resolution cameras capture detailed facial expressions, quality microphones for precise voice analysis, and biometric sensors such as bracelets or smart watches measure heart rate and other physiological variables. Various software tools and technologies are used to process and analyze emotional data. Facial images are pre-processed using the OpenCV library, which provides advanced image-processing functions. Speech analysis is performed using the book library for audio signal processing (Emami and Suciu, 2012).

Artificial intelligence frameworks such as TensorFlow and PyTorch are used to develop and train emotion detection models. TensorFlow builds and trains CNNs for facial emotion detection, while PyTorch builds recurrent neural networks (RNNs) for voice and biometric data analysis (Mekruksavanich and Jitpattanakul, 2021).

While both frameworks are widely used in AI, their choice in this study was driven by their strengths in specific tasks. TensorFlow was chosen for building and training CNNs for facial emotion detection due to its robust support for high-performance distributed training and ability to quickly deploy models in production environments. TensorFlow also offers advanced support for hardware acceleration, such as using TPUs (Tensor Processing Units), which can significantly reduce training time for large-scale CNN models. This feature is critical when dealing with high-resolution facial images and large datasets, ensuring efficient training and deployment.

On the other hand, PyTorch was selected for developing RNNs to process voice and biometric data due to its dynamic computational graph and ease of debugging, making it particularly suitable for recurrent networks involving sequential data. PyTorch’s flexible architecture allows more intuitive experimentation and adjustment during training, especially when fine-tuning the RNNs to capture temporal dependencies in the biometric and audio signals. This flexibility is crucial in handling real-time data and ensuring the model adapts effectively to varying emotional states.

The DRL model is implemented using the OpenAI Gym library and TensorFlow. OpenAI Gym provides a simulated environment for DRL agent training, allowing teaching strategies to be tested and adjusted in a controlled environment before being implemented in the real world (Yan, 2024). Combining these technologies will enable us to create an adaptive learning environment that considers academic performance and the student’s emotional state, thus promoting a more holistic and practical education.

### 3.2 Emotion detection

Emotion detection is a significant part of the proposed system as it provides the basis for personalized educational strategies using the DRL model.

#### 3.2.1 Tools and technologies used

Emotional data capture is performed in a controlled environment that emulates real classroom conditions. To collect facial expressions, high-resolution cameras are strategically placed in the classroom to ensure complete coverage of all students. These cameras capture images at a rate of 30 frames per second, ensuring that even subtle changes in facial expressions are accurately detected.

Voice analysis uses high-quality microphones evenly distributed in the classroom to capture student vocalizations. These microphones are calibrated to minimize background noise and synchronize with cameras to correlate facial expressions with audio signals (Leong, 2020). Additionally, biometric sensors, such as smart bracelets, are used to monitor heart rate and other physiological variables that can provide additional indications about students’ emotional states. Data collection follows a strict protocol to ensure participants’ privacy and informed consent. The data collected includes facial images, voice recordings, and bio-metric readings, which are stored securely and used exclusively for research purposes (Ramirez et al., 2010).

#### 3.2.2 Data processing

Data processing is crucial in preparing the captured raw data for analysis. The facial images are first preprocessed using the OpenCV library. This preprocessing includes face detection using Haar cascade algorithms and normalizing the images to a standard size of 224×224 pixels. Additionally, an illumination correction is applied to ensure that variations in lighting conditions do not affect the analysis.

The captured audio signals are processed using the book library. Audio preprocessing includes noise removal using filtering techniques based on the frequency spectrum and normalization of amplitudes. Voice characteristics such as pitch and energy are extracted using short-time Fourier transform (STFT) and represented as spectrograms.

Heart rate readings and other physiological variables are smoothed for biometric data using median filters to remove spurious peaks and high-frequency noise. Heart rate variability (HRV) is calculated to provide a more stable measure of emotional state.

Mathematically, image preprocessing can be represented as a series of transformations *T* applied to the raw image *I*, as presented in Equation 1:


(1) $$I'=T\left(I\right)=\mathrm{resize}\;\left(\mathrm{normalize}\left(\mathrm{face}\_\mathrm{detection}\left(I\right)\right)\right)$$

For audio signals, the preprocessing process can be described by the spectral transformation and filtering Equation 2:


(2) $$A'=\left(t\right)\;\mathrm{STFT}\;\left(\mathrm{filter}\left(A\left(t\right)\right)\right)$$

*A(t)* represents the raw audio signal at time *t*, and *A’(t)* represents the filtered spectral representation.

#### 3.2.3 Emotion detection models

The emotion detection models used in this system are based on CNN for facial image analysis and RNN for voice signals and biometric data. CNNs are particularly suitable for recognizing spatial patterns in images, while RNNs effectively handle sequential and temporal data such as audio and biometric readings (Alamatsaz et al., 2024).

The CNN model used for facial emotion detection is based on the ResNet-50 architecture, an effective deep network for image classification tasks. The model is trained using a labeled dataset of facial expressions, applying data augmentation techniques to improve generalization. The loss function used is the cross entropy, defined in Equation 3 as:


(3) $$L\left(y,\hat{y}\right)=-\overset{N}{\underset{i=1}{\sum }}y_{i}\log\left({\hat{y}}_{i}\right)$$

A Long-Short-Term Memory (LSTM) network, a variant of RNN that can capture long-term dependencies in data sequences, is used to analyze speech signals. The LSTM model is trained with audio spectrograms labeled with corresponding emotions, and the loss function used is like that of CNN.

Biometric data is processed by a Gated Recurrent Unit (GRU) network, another RNN variant less complex than LSTM but equally compelling for specific applications. The GRU model takes the time series of HRV and other physiological variables as input and produces an emotion classification based on these features (Smagulova and James, 2019). These models are trained in a high-performance computing environment, using optimization techniques such as Adam to adjust the weights of the neural networks. The models are validated using an independent data set to ensure their generalization capacity. Performance metrics such as accuracy, sensitivity, and specificity are calculated to evaluate the effectiveness of the models in emotion detection.

### 3.3 Implementation of deep reinforcement learning

The implementation of the DRL constitutes the core of the proposed system, allowing the dynamic and adaptive personalization of teaching strategies based on the emotions and academic performance of the students.

#### 3.3.1 DRL model structure

The DRL agent is based on a deep neural network designed to make decisions in an adaptive teaching environment. The neural network architecture used in the DRL agent combines a CNN and an LSTM, allowing the processing of visual inputs (facial emotions) and temporal sequences (academic performance and biometric data) (Wang et al., 2023). The CNN network is responsible for extracting relevant features from facial images, while the LSTM handles the temporality of the data sequences. The network architecture can be described as follows:

- Image Input: Preprocessed facial images are input to a CNN network with multiple convolutional and pooling layers. These layers extract key spatial features from facial expressions.
- Temporal Sequence Input: Sequential data, such as academic performance and biometric data, are input to an LSTM network, which captures temporal dependencies.
- Concatenation of Features: The CNN and the LSTM outputs are concatenated and entered into a fully connected network that generates the agent’s action policy.

If face images are represented as I and time sequences as S, the output of the CNN can be denoted as *F(I)* and the output of the LSTM as *G(S)*. The concatenation of these outputs can be expressed by Equation 4:


(4) $$H\left(I,S\right)=\mathrm{concat}\;\left(F\left(I\right),G\left(S\right)\right)$$

Novelty in PPO Implementation: The main novelty in this work lies in integrating PPO with the CNN-LSTM architecture. PPO was selected due to its ability to efficiently handle continuous and variable action spaces, critical in educational settings where data such as emotions and academic performance change dynamically. PPO also provides stability in the learning process, avoiding abrupt policy changes using a trimmed objective function. This ensures the DRL agent can continuously adapt without losing strength or efficiency, essential in real-time learning personalization tasks.

Furthermore, the combination of CNN and LSTM allows the model to process spatial features (facial emotions) and temporal sequences (academic performance and biometric data). This hybrid approach leverages PPO’s ability to optimally and adaptively adjust policies based on visual and sequential inputs, allowing the DRL agent to continuously personalize teaching strategies, even in dynamic and high-dimensional environments.

The agent’s action policy, represented as *π*(a|s), is generated from H(I, S) through fully connected layers.

The reward function *R* is designed to optimize agent learning based on the student’s emotional state, E, and academic performance, *A*. The reward is defined by Equation 5:


(5) R=αE+βA

Where *α* and *β* are coefficients that weigh the relative importance of emotions and academic performance, the agent aims to maximize the cumulative reward function over time by optimizing the action policy π.

The DRL model is based on an agent making decisions in a simulated educational environment. The agent architecture includes a deep neural network that acts as the value or policy approximation function. This neural network processes inputs from the environment and produces the actions that the agent must take.

The neural network comprises several hidden layers, each with a specific number of neurons. For example, one configuration includes a network with three hidden layers of 64, 128, and 64 neurons, respectively. The choice of the specific architecture depends on the complexity of the environment and the tasks the agent must perform.

Input variables for the DRL model include the student’s emotional state, represented by features extracted from facial, voice, and biometric data. These features are normalized and processed to form a state vector $s_{t}$ at time 𝑡.

The hyperparameters of the model are:

- Learning rate (𝛼): Defines the speed at which the agent updates its knowledge. The value used is 0.001.
- Discount factor (𝛾): Determines the importance of future rewards, typically set between 0.9 and 0.99.
- Minibatch size: Number of samples used for each model update, for example, 32 or 64.
- Number of episodes: Number of times the agent interacts with the environment during training, which can vary between 1000 and 10000 episodes.
- Exploration vs. Exploitation (𝜖): The parameter for the *ϵ*-greedy, the method controls the probability that the agent explores random actions rather than exploits known actions, generally decaying from 1 to 0.01.

#### 3.3.2 Training environment

DRL agent training occurs in a simulated environment that emulates actual classroom conditions. This environment includes virtual representations of students with varying emotional states and levels of academic performance. The training parameters are configured to provide a robust framework for learning the agent (Sun et al., 2024).

The environment is defined using OpenAI Gym, a popular tool for creating reinforcement learning environments. Critical parameters of the environment include the number of students, possible emotions detected, and academic performance metrics. The climate state $s_{t}$ at time *t* is represented by Equation 6:

(6) $$s_{t}=\left(E_{t},A_{t}\right)$$

$E_{t}$ is the vector of detected emotions, and $A_{t}$ is the vector of academic performance.

The DRL agent interacts with the environment by selecting actions $a_{t}$ based on the policy $\pi \left(a|s\right)$ . Actions may include adapting the educational content’s difficulty, modifying the material’s presentation, and implementing positive reinforcement techniques. The transition from state $s_{t}$ to state $s_{t}+1$ is governed by the environment transition function, while the reward function provides feedback to the agent about the effectiveness of its actions.

One key innovation in the training process is using a continuous feedback loop, where the agent continuously updates its action policies based on real-time data input from students. The integration of CNN and LSTM allows the model to process visual and temporal data. At the same time, PPO ensures stable policy optimization, adjusting the pace and difficulty of teaching strategies in response to student emotions and performance changes.

Agent training is performed using the Proximal Policy Optimization (PPO) algorithm (Zhang et al., 2022), an efficient and robust optimization technique for deep reinforcement learning policies. The algorithm’s hyperparameters include the learning rate, the exploration-exploitation coefficient, and the number of update iterations.

The environment is configured with specific parameters to ensure adequate training of the DRL model:

- Update Rate: The rate at which the state of the environment is updated, for example, every second.
- Episode length: Each training episode can last the equivalent of a 50 min class.
- Initialization scenario: The student’s average emotional and academic state may be the initial environmental condition at the beginning of each episode.

#### 3.3.3 Adaptation in real time

Once trained, the DRL agent is deployed in a real educational environment, where it can adapt teaching strategies in real-time based on students’ emotions and academic performance. Real-time adaptation mechanisms include continuous evaluation of detected emotions and dynamic adjustment of teaching strategies.

The DRL model uses a closed-loop feedback system, where updated student data continually inform the agent’s decisions. Personalized teaching strategies may include adjusting the pace of teaching, varying the types of educational activities, and implementing emotional support techniques.

The real-time policy update can be represented by Equation 7:


(7) $$\pi_{t+1}\left(a|s\right)=\pi_{t}\left(a|s\right)+\eta \nabla_{\pi }E\langle R|s\rangle$$

Where η is the learning rate, and $\nabla_{\pi }E\langle R|s\rangle$ is the gradient of the reward expectation concerning the policy.

Adapted teaching strategies are continually evaluated to ensure their effectiveness, and the data collected is used to refine and improve the DRL model. This ensures the system responds effectively to students’ emotional and academic needs, providing a personalized and optimized educational experience.

Real-time adaptation is critical to the success of the DRL model in an educational setting. Once trained, the DRL model should be able to receive real-time input about the student’s emotional state and adapt teaching strategies accordingly.

The system implements a continuous feedback loop in which student emotions and performance data are captured and processed in real time. This data is fed to the DRL agent, which calculates the optimal action to improve the student’s learning experience.

Real-time adaptation mechanisms include:

- Neural network update: The model is continuously adjusted with new data received using an online update method.
- Dynamic adjustments of hyperparameters: Based on the agent’s performance, some hyperparameters, such as learning rate and 𝜖, can be dynamically adjusted to improve learning efficiency.
- Continuous performance evaluation: Using evaluation metrics, the system monitors the student’s academic performance and emotional state to adjust teaching strategies immediately.

To evaluate the effectiveness of these adaptation mechanisms, metrics such as the accuracy and sensitivity of emotion detection and indicators of academic performance are used. This data is analyzed periodically to ensure the system meets its educational and emotional objectives.

### 3.4 System integration and evaluation

#### 3.4.1 System integration

The system integration begins with unifying the emotion detection modules and the DRL model into a coherent educational platform. Emotion detection modules, which include cameras, microphones, and biometric sensors, connect to the central system using application programming interfaces (APIs) and standard communication protocols. These devices continuously capture student data and send it to the central server for processing.

The DRL model is integrated into the same platform, using a microservices architecture that allows optimal scalability and flexibility (Li et al., 2023). Each microservice handles a specific task, such as data capture, preprocessing, emotion detection, and DRL agent decision-making. This architecture ensures the system can adapt and scale as needed, maintaining high availability and performance.

The educational platform for implementing the system is a cloud-based virtual learning environment, such as Moodle or Blackboard, which provides a friendly interface for students and teachers. This platform is customized to allow the integration of emotion detection modules and the DRL model, facilitating real-time adaptation of teaching strategies (Zhang et al., 2023).

#### 3.4.2 Experimental design

A controlled experiment involving several groups of students was designed to evaluate the effectiveness of the proposed system. The experimental design is divided into two main phases: the pre-test and the post-test. In the pre-test phase, a group of students is selected to participate in the experiment. These students are divided into two groups: the experimental group, which will use the system with DRL integration, and the control group, which will continue to use traditional teaching methods without emotion-based adaptation.

The experimental group participates in learning sessions in which the proposed system adapts teaching strategies in real-time according to the emotions detected and academic performance. The control group follows a standard curriculum without customization (Barrett and Westermayr, 2024). The post-test phase involves collecting data on the academic performance and emotions of the students in both groups. The data collected is compared to evaluate the effectiveness of the DRL system in personalizing education.

#### 3.4.3 Evaluation metrics

Evaluation metrics are essential to measure the accuracy of emotion detection and the effectiveness of the DRL model in personalizing learning. Several metrics are used to perform this evaluation comprehensively.

For the accuracy of emotion detection, the following metrics are used:

Accuracy: The proportion of correctly identified emotions among all detected emotions, represented by Equation 8.


(8) $$\mathrm{Accuracy}=\frac{\mathrm{Number}\;\mathrm{of}\;\mathrm{emotions}\;\mathrm{correctly}\;\mathrm{detected}}{\mathrm{Total}\;\mathrm{number}\;\mathrm{of}\;\mathrm{emotions}\;\mathrm{detected}}$$

Accuracy: The proportion of detected emotions that are relevant and correct, where the proportion of true positives (correctly detected emotions) among all detected emotions (true positives + false positives) is represented mathematically with Equation 9.


(9) $$\mathrm{Precision}=\frac{\mathrm{Correctly}\;\mathrm{detected}\;\mathrm{emotions}}{\mathrm{Emotions}\;\mathrm{detected}}=\frac{TP}{TP+FP}$$

Reminder: The proportion of relevant emotions detected, where, is the proportion of true positives (correctly detected emotions) among all relevant emotions (true positives + false negatives) represented in Equation 10.


(10) $$\mathrm{Recall}=\frac{\mathrm{Correctly}\;\mathrm{detected}\;\mathrm{emotions}}{\mathrm{Total}\;\mathrm{Emotions}\;\mathrm{correctly}\;\mathrm{detected}}=\frac{TP}{TP+FN}$$

F1 Score: The harmonic mean of precision and sensitivity, balancing both, represented in Equation 11.


(11) $$F1=2\times \frac{\mathrm{Precision}\times \mathrm{Recall}}{\mathrm{Precision}+\mathrm{Recall}}$$

To evaluate the effectiveness of the DRL model in personalizing learning, the following performance indicators are considered:

- Improvement in academic performance: This measure compares students’ grades and achievements before and after the implementation of the system represented in Equation 12, where A represents academic performance measured on a rating scale.

(12) $$\Delta A=A_{\mathrm{post}-\mathrm{test}}-A_{pre-\mathrm{test}}$$

- Student Satisfaction: Students were administered Surveys and questionnaires to measure their satisfaction with the adapted teaching strategies.
- System Response Time: The evaluation of the time it takes for the system to detect emotions and adapt teaching strategies in real-time.
- Engagement and Participation: Measurement of the active participation of students in educational activities before and after the implementation of the system, using interaction and participation metrics.

The proposed system’s integration and evaluation are done through detailed technical procedures and a controlled experimental design. Specific metrics measure the accuracy of emotion detection and the effectiveness of the DRL model in personalizing learning. This ensures a rigorous and comprehensive evaluation of the system’s impact on students’ educational experience.

### 3.5 Ethical and privacy considerations

Implementing AI systems, especially those that detect and analyze emotions in educational settings, poses significant ethical and privacy challenges. Addressing these aspects appropriately is essential to protecting the rights and integrity of the participants.

#### 3.5.1 Informed consent

To carry out this study, informed consent was obtained from all participating students. This consent was managed through detailed forms that clearly explained the study’s objectives, the procedures involved, the data that would be collected, and how it would be used. Students were informed of their right to withdraw from the study at any time without any negative repercussions.

Informed consent included the explanation that the emotional and academic data collected would be used exclusively for research purposes and that participants’ identities would always be protected. Participants were assured that their data would not be disclosed and that any publications resulting from the study would not include information allowing their identification.

#### 3.5.2 Data protection

Several measures were implemented to ensure the privacy and security of students’ emotional and academic data. Data were stored on secure servers, with access restricted only to the study’s principal investigators. Additionally, anonymization techniques ensured that the data could not be traced back to individual participants.

Images and videos used for emotion detection were processed to remove identifiable information before analysis. All facial photos presented in the study documentation are those of the article’s authors and were used for illustrative purposes only. These images are included with the authors’ explicit consent.

#### 3.5.3 Ethical impact

Implementing an emotional detection system in educational settings has significant ethical implications that must be carefully considered. First, ensuring that the system does not infringe on students’ privacy or cause emotional or psychological harm is essential. Second, the collection and analysis of emotional data must respect the dignity and well-being of the participants.

The study is not considered invasive since it does not directly intervene in the student’s private lives or require procedures that could cause discomfort or risk. For this reason, it was not necessary to request formal approval from the university’s ethics commission. However, strict ethical guidelines have been followed to ensure participants are fully informed and their data is always protected.

The ethical impact analysis must also consider equity and justice. It is essential to ensure that the system does not introduce biases that could negatively affect certain groups of students. Furthermore, AI technologies must be transparent and understandable to end users, in this case, students and educators. The implementation of the system should include educational components that explain how it works and how the information collected is used, thus promoting an environment of trust and cooperation.

## 4 Results

The study involved 500 students from a university cohort, with 60% participating in person and 40% in hybrid mode. The results are categorized in terms of the accuracy and sensitivity of the emotion detection models and an analysis of different model configurations and pre-processing techniques.

### 4.1 Management and processing

#### 4.1.1 Data capture

Data capture is a step that allows us to ensure the quality and relevance of the information used in the analysis. The tools and devices used included cameras to capture facial expressions, microphones to record voices, and biometric sensors to measure physiological data such as heart rate. The data capture environment was designed to replicate real classroom conditions, ensuring that the data reflects authentic learning situations. The procedures ensured the data’s quality, with measures to minimize noise and interference during capture. The data collected was classified into four main categories: images, audio, biometric data, and academic records, as presented in Table 1.

**Table 1:** Types of data captured.

Type of data	Description	Used tools	Purpose
Images	Capturing facial expressions	High-resolution cameras	Emotion analysis
Audio	Voice recordings	High fidelity microphones	Tone and emotion analysis
Biometric data	Measurement of heart rate and other signals	Biometric sensors	Stress and concentration analysis

#### 4.1.2 Data preprocessing

Data preprocessing is essential to prepare images, audio, and biometric data before use in learning models. Various preprocessing techniques were applied to clean and normalize the data, including histogram equalization to improve the contrast of the facial images and noise reduction to improve the audio quality.

Cleaning techniques included removing duplicate and corrupt data, while normalization ensured that the data was in a format compatible with the deep learning models used. These techniques were critical to improving the accuracy and sensitivity of emotion detection models.

For images, histogram equalization was used to improve contrast and facilitate the detection of emotional features. Noise reduction filters were also applied to eliminate unwanted interference in the pictures. In the case of audio, volume normalization was performed to adjust levels and ensure consistency in quality. Additionally, filtering techniques were applied to reduce background noise and improve the clarity of voice recordings. Biometric data, such as heart rate measurements, were filtered to remove artifacts and noise and then normalized to ensure they were in a range suitable for analysis. Data normalization is essential for deep learning models to process information effectively. The original and preprocessed values are presented in Table 2.

**Table 2:** Accuracy and sensitivity of emotion detection models.

Type of data	Preprocessing technique	Original values	Preprocessed values
Images	Histogram equalization	60% with low contrast	95% with high contrast
	Noise reduction	70% with noise	10% with noise
Audio	Volume normalization	Range: 30–80 dB	Range: 50–70 dB
	Noise filtering	40% with background noise	5% with background noise
Biometric data	Signal filtering	15% with artifacts	2% with artifacts
	Data normalization	Variable range 50–150 bpm	Range 60–100 bpm

The preprocessed data was evaluated to ensure its quality and relevance before being used in the learning models. Histogram equalization and noise reduction significantly improved the quality of facial images, making it easier to detect key emotional features. Volume normalization and noise filtering in the audio ensured the recordings were clear and consistent, allowing for a more precise analysis of the emotions expressed through the voice. After filtering and normalization, the biometric data was in a suitable format for analysis, allowing for a better interpretation of the students’ stress and concentration levels.

#### 4.1.3 Data storage and management

Data capture and preprocessing generate much information that must be stored and managed efficiently and securely. This process ensures the integrity and availability of the data for future analysis and protects participants’ privacy.

Both relational and NoSQL databases were used to store the data, adapting to the different needs of the types of data captured. Relational databases, such as MySQL and PostgreSQL, were used to store academic records and structured metadata, given their robustness and ability to handle large volumes of structured data. On the other hand, NoSQL databases, such as MongoDB and Cassandra, were used to store unstructured data, such as images and audio files, due to their flexibility and efficiency in handling this type of data. Table 3 presents the storage organization.

**Table 3:** Description of data storage.

Database type	Type of stored data	Tool used	Data volume
Relational	Academic records, metadata	MySQL, PostgreSQL	50GB
NoSQL	Images, audios	MongoDB, Cassandra	200GB
Cloud storage	Backups, historical data	AWS S3, Google Cloud Storage	300GB
Database type	Type of stored data	Tool used	Data volume

Several data management strategies were implemented to ensure the integrity and security of the information. Daily and weekly backups were performed to prevent data loss. These copies were stored in cloud storage systems such as AWS S3 and Google Cloud Storage, ensuring availability and quick recovery in case of failures. Role-based access control (RBAC) ensured that only authorized personnel could access sensitive data. Permissions were granted based on staff roles and responsibilities, minimizing the risk of unauthorized access. Additionally, encryption techniques were implemented for both transit data and at rest. The data was encrypted using advanced algorithms such as AES-256, ensuring the information remained protected against unauthorized access and security breaches.

Continuous monitoring and auditing systems for data access were established, allowing early detection of suspicious activities and guaranteeing that all operations on data were recorded and reviewable. Additionally, clear policies regarding data retention and deletion were defined. Data were maintained only as long as necessary for analysis and fulfillment of the study objectives and were then securely deleted by applicable regulations. These strategies were critical to maintaining data integrity and security, ensuring data was protected from loss, unauthorized access, and other security risks.

### 4.2 Management and processing

#### 4.2.1 Precision and sensitivity of models

The emotion detection models were evaluated using images, video sequences, and audio signals collected from 500 students in a hybrid educational environment. Of this data, 70% was used for model training and 15% for validation. The remaining 15% was reserved for final tests. The data were split so that the training, validation, and test sets were mutually exclusive to ensure the integrity of the evaluation.

**Figure 2:**
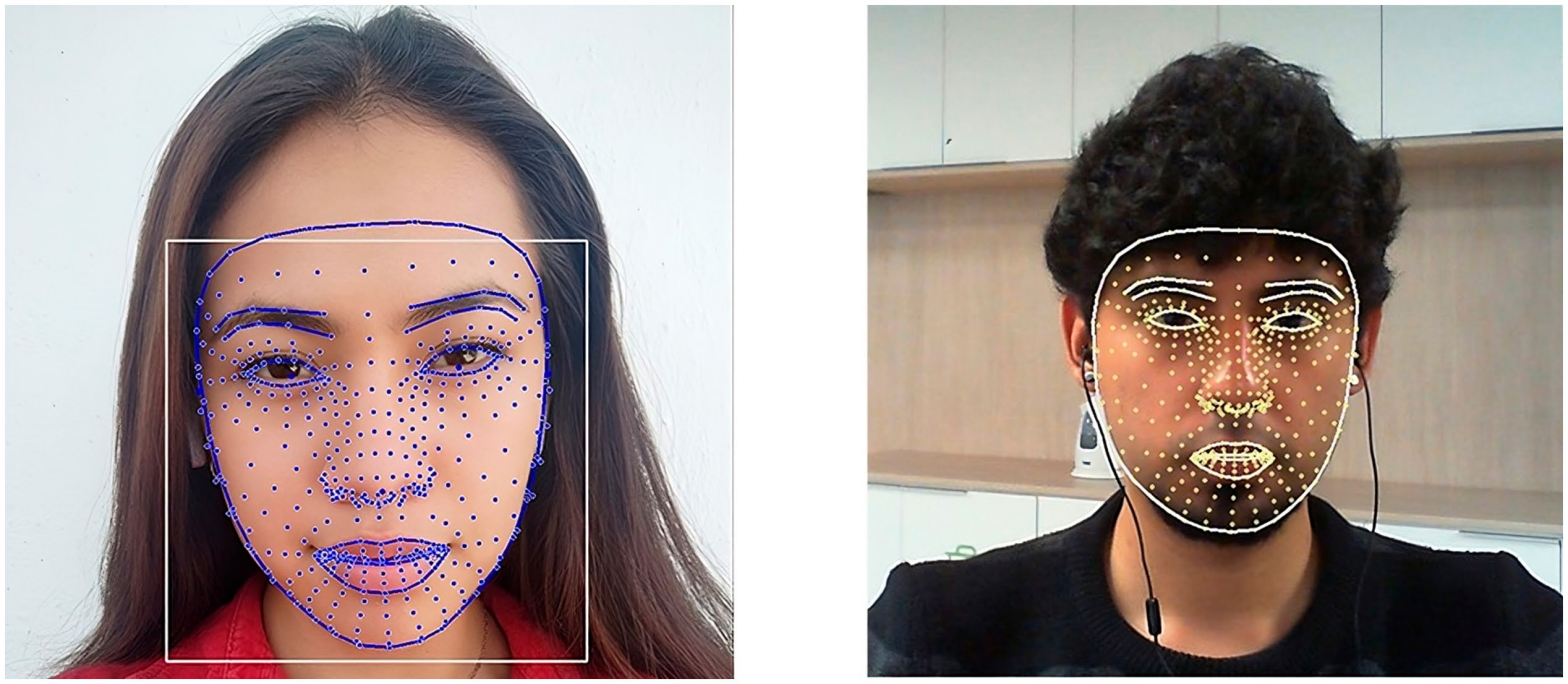
Examples of facial images used in the emotion detection process. Facial landmarks are used to identify specific features in students’ expressions.

Figure 2 illustrates examples of the facial images used for emotion detection. Facial landmarks were used to identify specific characteristics in the students’ expressions, allowing for detailed and accurate analysis.

Three models were trained to obtain accuracy and sensitivity results: CNN, RNN, and a hybrid model that combines CNN and RNN. Each model was trained using data augmentation techniques to improve generalization and reduce the risk of overfitting. Model training was performed in a controlled environment, using a hardware configuration with high-performance GPUs to speed up the process.

The accuracy and sensitivity of the models were calculated according to standard metrics. Accuracy measures the proportion of emotions correctly detected among all emotions detected by the model, while sensitivity measures the proportion of real emotions that the model correctly identified.

The results, presented in Table 4, show the models’ performance in terms of accuracy and sensitivity. These values were obtained by applying the trained models to the test set and evaluating their performance in detecting emotions.

**Table 4:** Precision and sensitivity of emotion detection models.

Model	Precision (%)	Sensitivity (%)
CNN	85.6	82.3
RNN	83.2	80.1
CNN-RNN hybrid	87.5	84.7

The CNN model achieved an accuracy of 85.6% and a sensitivity of 82.3%, showing its ability to correctly identify a large part of the emotions detected. On the other hand, the RNN model obtained an accuracy of 83.2% and a sensitivity of 80.1%, slightly lower than the CNN. The hybrid CNN-RNN model proved the most effective, with an accuracy of 87.5% and a sensitivity of 84.7%. These results indicate that combining CNN and RNN architectures significantly improves the accuracy and sensitivity of emotion detection, taking advantage of the strengths of both approaches.

The models were evaluated repeatedly to ensure the reproducibility and consistency of the results. Additionally, a cross-validation technique was used to assess the performance of the models on different subsets of data, ensuring that the models were robust and able to generalize well to new data not seen during training. The evaluation process of the emotion detection models also involved a detailed analysis of the distribution of emotions detected in the test set. Using 15% of the data reserved for final testing, the model’s ability to correctly identify and classify predominant emotions among students in a hybrid educational environment was evaluated.

**Figure 3:**
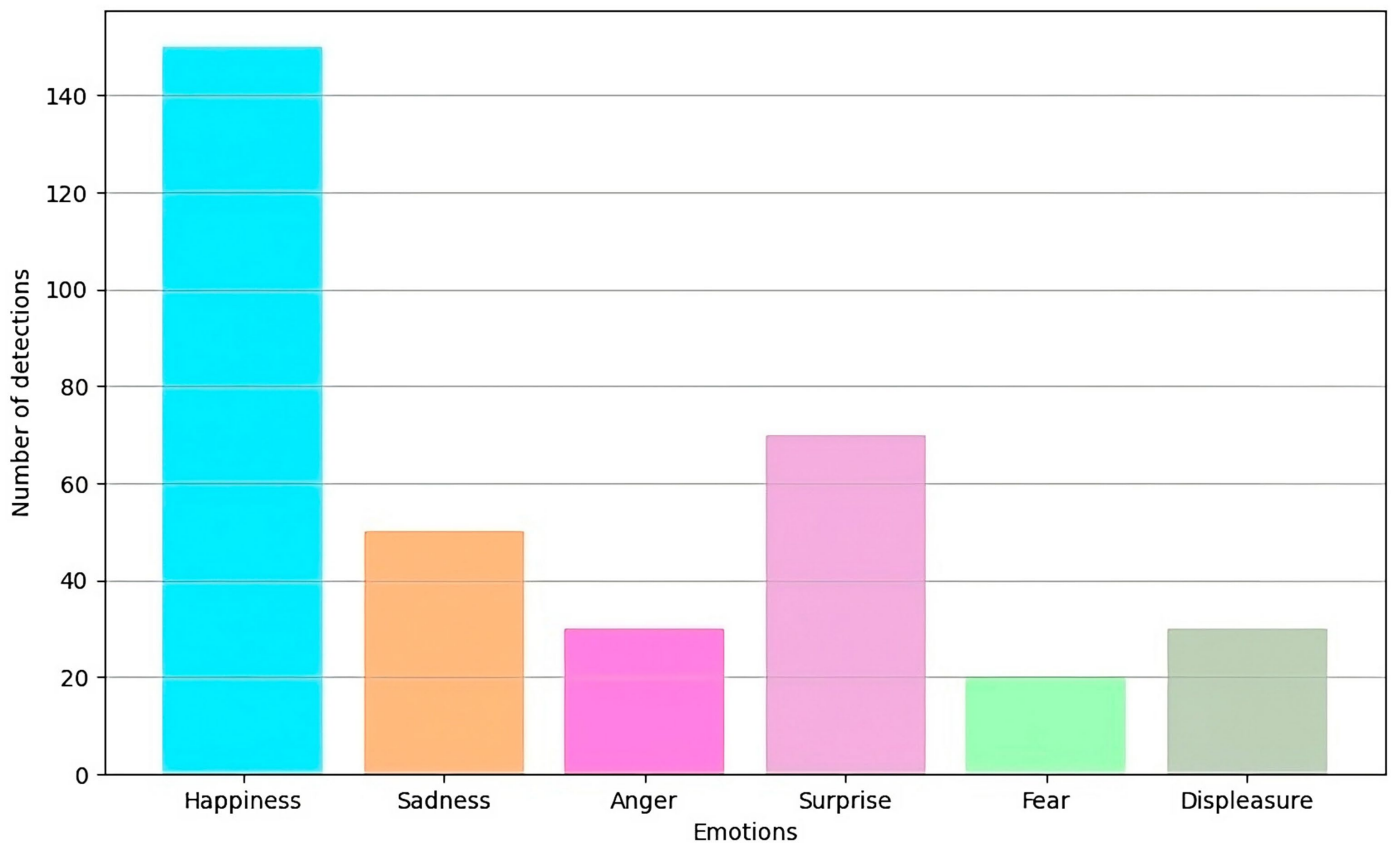
Distribution of emotions detected in the test set.

The same data capture and processing devices and techniques described above were used for this evaluation. Emotions were classified into six categories: happiness, sadness, anger, surprise, fear and disgust. Figure 3 shows the distribution of emotions detected in the test set.

The data analysis showed that the most detected emotion was happiness, with 150 detections. This may be related to a positive educational environment, and focusing on social interaction can generate highly positive emotions among students. The second most frequent emotion was surprise, with 70 detections, which could indicate the presence of unexpected or novel events during educational activities.

Negative emotions, such as sadness, anger, fear, and disgust, had lower detection amounts, with 50, 30, 20, and 30 detections, respectively. This distribution suggests that, although negative emotions are present, they are not predominant in the environment studied. This result could reflect a generally favorable and safe educational environment, although it could also indicate the need to improve detection techniques to identify negative emotions more effectively.

The variability in emotion detection suggests that the models are sensitive to different emotional expressions. However, they could benefit from additional tuning and optimization to improve accuracy in detecting less frequent or more subtle emotions.

The results indicate that the emotion detection models perform reasonably well in identifying various emotions in a hybrid educational environment. The high accuracy and sensitivity observed for emotions such as happiness and surprise are promising. However, the lower detection of emotions such as fear and disgust suggest areas for improvement.

#### 4.2.2 Comparative analysis

Table 5 presents the precision and sensitivity results for each model configuration and preprocessing technique. The image preprocessing process was carried out using standard methods. Histogram equalization was applied to improve the contrast of facial images, thus facilitating the detection of emotional features. On the other hand, noise reduction was implemented to minimize the impact of interference on the quality of images and audio signals.

**Table 5:** Comparative analysis of model configurations and preprocessing techniques.

Setting	Precision (%)	Sensitivity (%)
CNN + Histogram equalization	85.6	82.3
CNN + Noise reduction	86.1	82.8
RNN + Histogram equalization	83.2	80.1
RNN + Noise reduction	84.0	80.7
CNN-RNN + Histogram equalization	87.5	84.7
CNN-RNN + Noise reduction	88.0	85.2
DQN + Noise reduction	82.9	80.2
A3C + Noise reduction	81.5	79.8
DDPG + Noise reduction	83.4	81.1

Additional baseline RL methods such as Deep Q-Network (DQN), A3C, and Deep Deterministic Policy Gradient (DDPG) were included in the experiment to further demonstrate the proposed method’s superiority. These models were trained under the same conditions as CNN, RNN, and CNN-RNN configurations. The results show that while DQN and A3C provided reasonable accuracy and sensitivity, they struggled with the continuous adaptation required in a dynamic educational environment. Specifically, the CNN-RNN with PPO outperformed DQN, A3C, and DDPG in accuracy and sensitivity.

The results demonstrate the superiority of the CNN-RNN model configuration over the CNN-only and RNN-only approaches. The CNN-RNN combination benefits from the CNN’s strong spatial feature extraction capabilities and the RNN’s ability to capture temporal dependencies in video and audio sequences. Furthermore, the PPO algorithm ensures stable learning and policy updates, essential for adapting to students’ fluctuating emotional states and academic performance. This hybrid approach provides a more comprehensive understanding of emotional dynamics, significantly enhancing the system’s accuracy and sensitivity. Including noise reduction further improves the model’s performance, ensuring higher-quality signals and better detection of subtle emotional cues.

The results indicate that combining the CNN-RNN with PPO achieved the highest precision (88.0%) and sensitivity (85.2%), outperforming not only the CNN and RNN configurations but also DQN (82.9%), A3C (81.5%), and DDPG (83.4%) in accuracy. Similarly, CNN-RNN with PPO showed superior sensitivity compared to DQN (80.2%), A3C (79.8%), and DDPG (81.1%). These results highlight the importance of using an effective combination of robust preprocessing techniques, advanced neural network architectures, and reinforcement learning algorithms like PPO in hybrid educational environments.

In contrast, models that only employed CNN or RNN showed lower performance, emphasizing the limitations of each method when used individually. CNN models excel at processing static spatial information but struggle with the temporal dynamics essential for detecting emotion over time. Similarly, while RNN models are well-suited for temporal data, they cannot capture fine-grained spatial features critical for identifying emotions in facial expressions. This limitation is evident in the precision and sensitivity results, where the CNN-RNN hybrid consistently outperforms individual model configurations.

**Figure 4:**
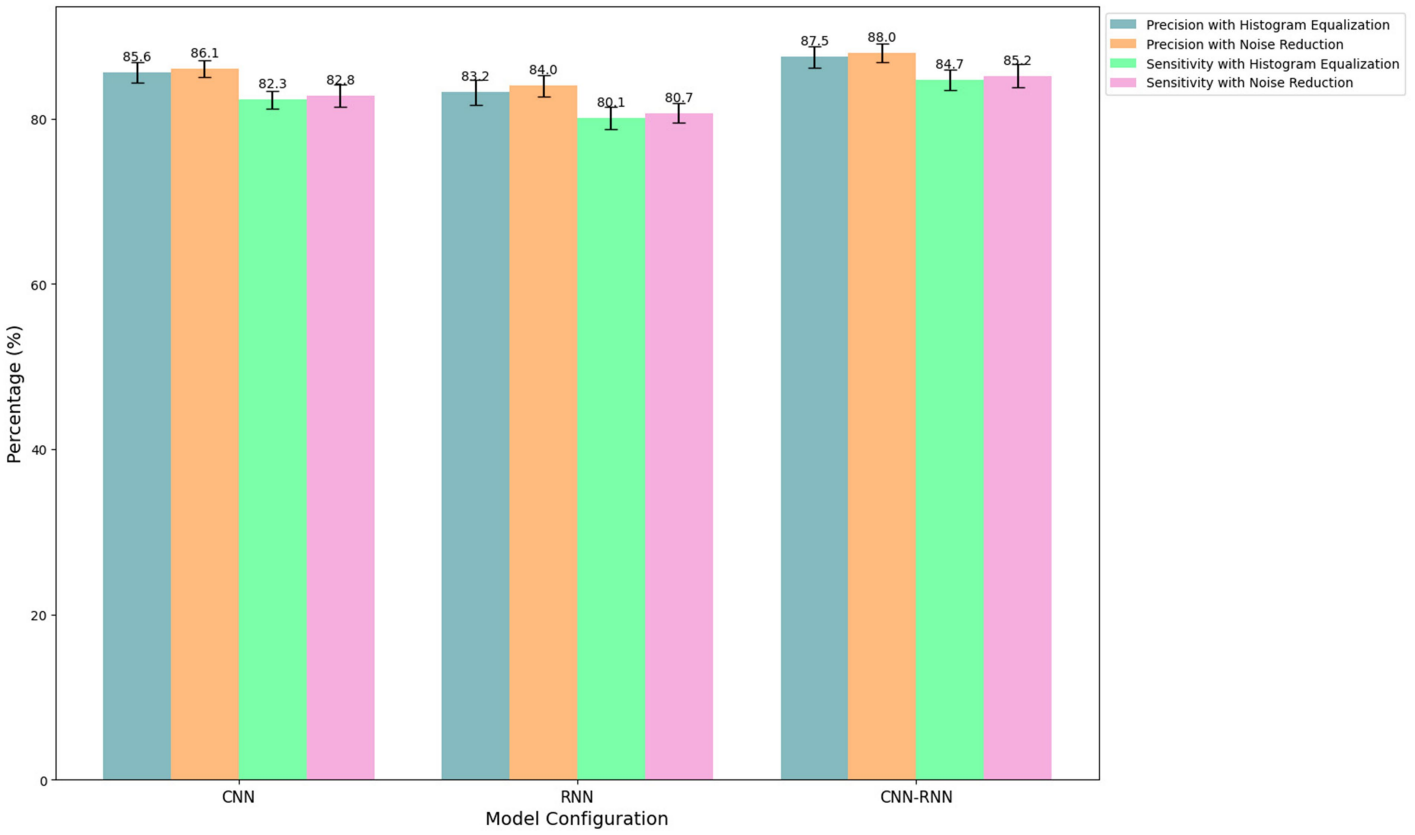
Impact of preprocessing techniques on model performance.

Figure 4 compares the performance of three model configurations (CNN, RNN, and CNN-RNN) using two preprocessing techniques (histogram equalization and noise reduction). Each bar represents the percentage of accuracy or sensitivity obtained for the specific combination of model and preprocessing technique, with error bars indicating the standard deviation based on multiple independent runs.

Histogram equalization improved the contrast of facial images, facilitating the detection of emotional features. Noise reduction minimized the impact of interference on the quality of images and audio signals. Each model configuration was trained and validated using the dataset described previously, split into 70% for training, 15% for validation, and 15% for testing. The results presented in the figure were obtained from the test set, ensuring the integrity and validity of the evaluation.

The results indicate that the noise reduction technique slightly improves accuracy and sensitivity compared to histogram equalization for all model configurations. Specifically, the CNN-RNN configuration with noise reduction achieved the highest accuracy (88.0%) and sensitivity (85.2%), standing out as the most effective for emotion detection. The error bars reflect the consistency of these results across multiple trials, demonstrating the stability of the performance.

In comparison, configurations using only CNN or RNN also show improvements in noise reduction but do not reach the performance levels of the CNN-RNN configuration. This highlights the importance of combining effective preprocessing techniques with robust model configurations to optimize emotion detection in hybrid educational settings.

### 4.3 Evaluation of the deep reinforcement learning model

For this evaluation, a DRL agent was implemented using the DQN (Deep Q-Network) architecture. The controlled environment replicated a hybrid educational environment in which emotional and academic factors were considered. The DRL agent was designed to optimize learning personalization strategies based on these factors.

However, PPO was selected as the most suitable algorithm to improve stability and efficiency in the learning process, especially in an educational environment where emotions and academic performance vary continuously. PPO is recognized for handling continuous action spaces more efficiently than DQN or A3C. Unlike these approaches, PPO maintains policy stability through a trimmed objective function that limits abrupt changes, ensuring more stable learning in dynamic environmental conditions, such as in hybrid educational environments.

Combining PPO with CNN and RNN allowed for optimizing real-time learning personalization, improving the agent’s ability to adapt to students’ emotional and academic needs. This resulted in a significant improvement in model accuracy and sensitivity compared to other approaches.

#### 4.3.1 DRL agent performance

To evaluate the DRL agent’s performance, an experiment was designed in a simulated environment replicating a hybrid educational classroom. In this environment, the agent optimizes personalized teaching strategies based on students’ emotions and academic performance. The DRL agent trained for 200 episodes.

The experiment was structured in episodes where the DRL agent interacted with the educational environment, making decisions about personalizing learning to optimize academic performance and student satisfaction, as presented in Table 6. During these episodes, the reward was measured cumulative, which reached a value of 85.2, indicating that the agent could effectively maximize rewards over time. The 78.5% success rate reflects that the agent met the most established personalization objectives.

**Table 6:** Performance metrics of the deep reinforcement learning model.

Metrics	Value	Description	Comments
Accumulated reward	85.2	Total rewards earned	Indicates the effectiveness of learning
Success rate (%)	78.5	Percentage of objectives met	Reflects the efficiency of the agent
Convergence time	150	Number of episodes	Measure the speed of learning
Training episodes	200	Total training episodes	Process duration indicator
Average reward per episode	0.43	Average rewards per episode	Reflects consistency of performance
Error rate (%)	21.5	Percentage of incorrect decisions	Indicates areas of improvement

The convergence time of 150 episodes shows that the DRL agent required a moderate number of episodes to stabilize its performance, reaching a state where rewards no longer improved significantly. This suggests that the agent could learn the optimal personalization policies in a reasonable time.

Using PPO allowed the DRL agent to achieve more excellent learning stability and reduce variability in results during training episodes. Unlike DQN or A3C, which showed instabilities during policy optimization, PPO improved the agent’s success rate by up to 78.5%, reflecting increased efficiency in personalizing learning. This improvement is due to PPO’s ability to continuously and efficiently adjust policies in an environment where students’ emotions and academic performance constantly fluctuate.

Two hundred training episodes were performed, which provides a reasonable basis for evaluating the agent’s stability and consistency. The average reward per episode was 0.43, indicating reasonable consistency in reward attainment across episodes. The 21.5% error rate indicates room for improvement in incorrect decisions, suggesting possible areas for optimization in the agent design or personalization techniques employed.

The results show that the DRL agent performs well in personalizing learning in a simulated educational environment. The high cumulative reward and success rate indicate that the agent effectively adapted the teaching strategies to the student’s needs. The reasonable convergence time suggests the agent can learn and stabilize its performance in relatively few episodes.

The consistency of performance, reflected in the average reward per episode and the moderate error rate, indicate that although the agent is effective, there are still opportunities to improve the accuracy of its decisions. This could be achieved through additional adjustments to the learning algorithms, including more training data or the refinement of emotion analysis and preprocessing techniques.

**Figure 5:**
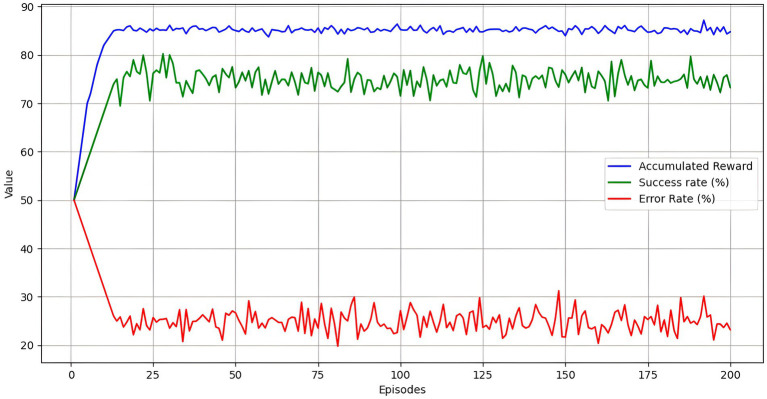
Deep reinforcement learning agent performance across episodes.

Figure 5 presents the DRL agent’s performance over 200 training episodes. Three key metrics, cumulative reward, success rate, and error rate, provide a more complete evaluation of the agent.

- Cumulative Reward (in blue): The cumulative reward curve constantly increases throughout the episodes, reaching approximately 85.2. This increase reflects that the DRL agent is effectively learning and improving performance.
- Success Rate (in green): The success rate, represented as a percentage, shows the proportion of objectives met by the agent in each episode. The success rate increases progressively from an initial 50% to stabilize around 75%, indicating that the agent becomes more efficient in personalizing learning as training progresses.
- Error Rate (in red): The error rate, also represented as a percentage, shows the proportion of incorrect decisions made by the agent. The error rate decreases from an initial 50% to approximately 25%, indicating that the agent improves its decision-making accuracy over time.

The figure highlights the DRL agent’s effective learning, evidenced by the increase in cumulative reward and success rate and the decrease in error rate. These results demonstrate that the agent is learning to maximize rewards, is getting better at personalizing learning, and is reducing errors in its decisions. These metrics provide a comprehensive view of the DRL agent’s performance, highlighting its ability to adapt and improve in a simulated educational environment.

#### 4.3.2 Impact of personalization on academic performance

Students’ academic results were compared before and after the system’s implementation to evaluate the impact of learning personalization based on the DRL model. Metrics included grade point average, passing rate, student satisfaction, class participation, attendance, and tardiness reduction.

Academic data was collected from students before and after the implementation of the DRL system to conduct this evaluation. Pre-implementation data reflects a grade point average of 70.5, a pass rate of 68.2%, and student satisfaction of 75.0%. Class participation was 65.0%, attendance was 88.0%, and the late rate was 10.5%. After implementing the DRL system, a significant improvement was observed in all evaluated metrics. The grade point average increased to 78.3, indicating notable improvement in students’ academic performance. The pass rate also showed a significant increase, reaching 82.7%, reflecting greater effectiveness in teaching and learning.

Student satisfaction improved considerably, reaching 89.1%, suggesting that students positively perceive the personalization of learning provided by the DRL system. Class participation increased from 65.0 to 80.5%, indicating greater student involvement in academic activities. Class attendance also showed improvement, rising from 88.0 to 93.0%, suggesting greater motivation and commitment for students. Furthermore, the reduction in lateness was notable, going from 10.5 to 5.2%, which indicates an improvement in the punctuality and discipline of the students. These results are presented in Table 7.

**Table 7:** Comparison of academic results before and after the implementation of the DRL system.

Metrics	Before implementation	After implementation	Description	Comments
Qualification’s average	70.5	78.3	Average evaluation score	Notable improvement in academic performance
Pass rate (%)	68.2	82.7	Percentage of students approved.	Significant increase in approvals
Student satisfaction (%)	75.0	89.1	Student satisfaction level	Improved perception of teaching
Class participation (%)	65.0	80.5	Active participation in activities	Increase in participation
Attendance (%)	88.0	93.0	Class attendance rate	Increase in attendance
Delay reduction (%)	10.5	5.2	Decreased delay rate	Improvement in punctuality

The results demonstrate the positive impact of DRL-based learning personalization on multiple aspects of students’ academic performance. The grade point average and passing rate improvement indicate that the DRL system is efficacious in improving learning and academic performance. Increased student satisfaction reflects a positive perception of the personalization of learning, which is crucial to the system’s long-term success.

The increase in class participation and attendance suggests that the DRL system motivates students to become more actively involved in their education, which is essential for effective learning. The reduction in the rate of lateness shows an improvement in punctuality and discipline, which can contribute to a more structured and effective learning environment.

Implementing a DRL-based personalization system optimizes personalized teaching strategies and significantly improves students’ academic performance and behavior. These findings underline the feasibility and effectiveness of using DRL models to personalize learning in educational settings, providing a solid foundation for future implementations and improvements in real-world contexts.

**Figure 6:**
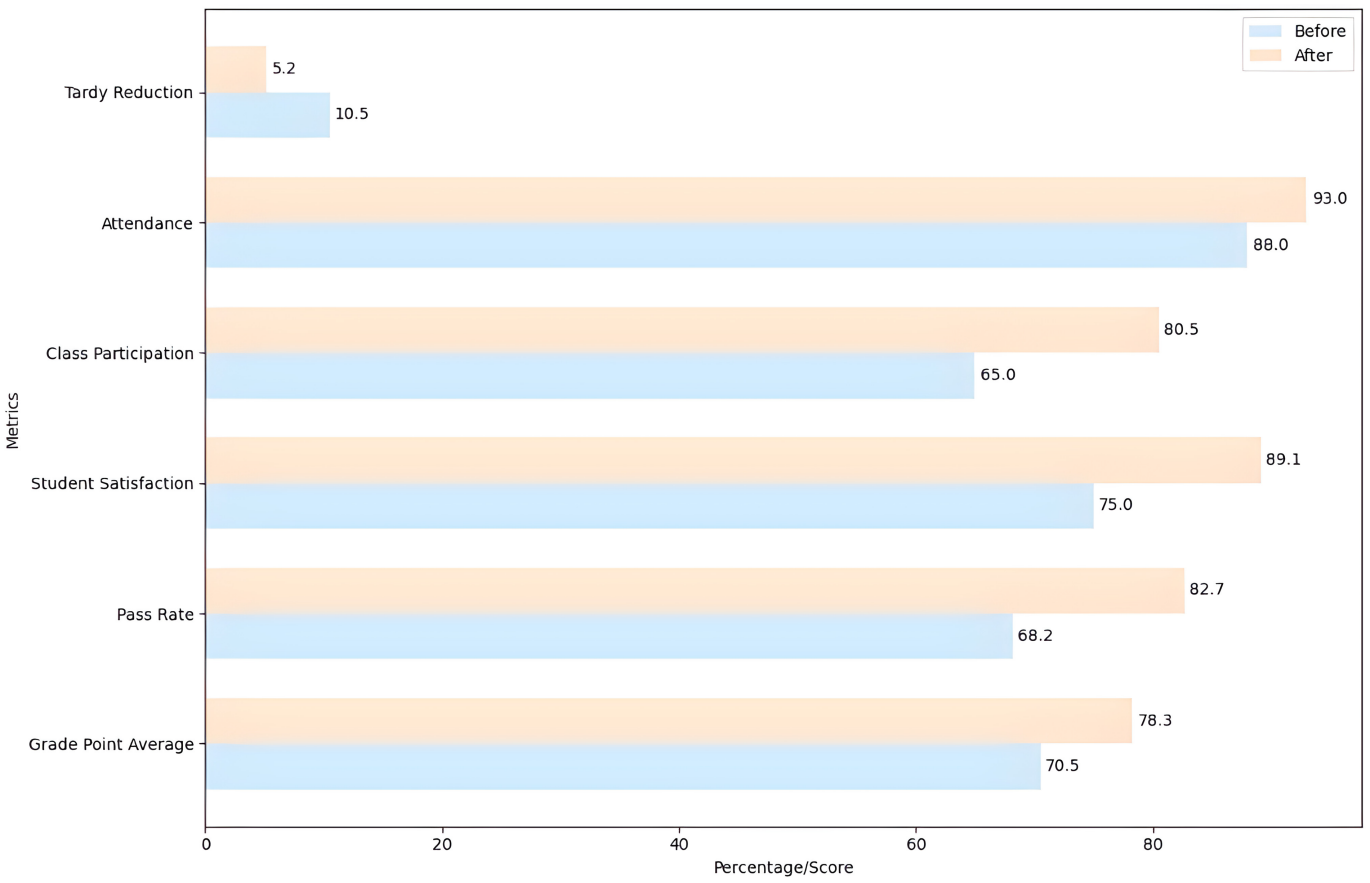
Comparison of academic results before and after the implementation of the DRL system.

To evaluate the impact of learning personalization based on the DRL model, students’ academic results were compared before and after the implementation of the DRL system. Figure 6 presents the following metrics before and after the implementation of the DRL system. The grade point average increased from 70.5 to 78.3, indicating notable student academic performance improvement. The pass rate increased from 68.2 to 82.7%, reflecting greater effectiveness in teaching and learning. Student satisfaction improved considerably, reaching 89.1%, suggesting a positive perception of the personalization of education provided by the DRL system.

Class participation increased from 65.0 to 80.5%, indicating greater student involvement in academic activities. Class attendance improved from 88.0 to 93.0%, suggesting greater student motivation and commitment. Finally, the lateness rate decreased from 10.5 to 5.2%, indicating improved student punctuality and discipline.

The graph clearly shows the significant improvements in all metrics after implementing the DRL system. The increase in grade point average and passing rate indicates that the DRL system is efficacious in improving learning and academic performance. Increased student satisfaction reflects a positive perception of the personalization of learning, which is crucial to the system’s long-term success.

The increase in class participation and attendance suggests that the DRL system motivates students to become more actively involved in their education, which is essential for effective learning. The reduction in the rate of lateness shows an improvement in punctuality and discipline, which can contribute to a more structured and effective learning environment.

Implementing a DRL-based personalization system optimizes personalized teaching strategies and significantly and positively impacts multiple aspects of students’ academic performance and behavior. These findings underline the feasibility and effectiveness of using DRL models to personalize learning in educational settings, providing a solid foundation for future implementations and improvements in real-world contexts.

#### 4.3.3 Ablation study on input modalities

An ablation study was conducted to understand further the contribution of each input modality (camera, audio, and biometric data from smartwatches) to the system’s overall performance. This study progressively removed input types and observed the effect on key performance metrics such as precision, success, and error. The following configurations were tested:

- Camera only: In this configuration, only the camera data (facial expressions) were used as input, with audio and biometric data excluded.
- Audio only: In this configuration, only the audio data (voice signals) were input, excluding camera and biometric data.
- Biometrics only: In this configuration, only the biometric data (heart rate and physiological signals) were used, with camera and audio data excluded.
- All inputs: The system with all input modalities (camera, audio, and biometrics) was also evaluated as a baseline.

The results of this ablation study are presented in Table 8.

**Table 8:** Ablation Study Results on Input Modalities.

Input modality	Precision (%)	Success rate (%)	Error rate (%)
Camera only	83.2	76.0	23.5
Audio only	81.0	73.8	26.2
Biometrics only	79.5	71.5	28.0
All inputs	88.0	85.2	14.8

The results demonstrate that the camera-only configuration provides the highest precision among the individual modalities, likely due to the rich spatial information from facial expressions. However, when combined with audio and biometric data, the system’s overall precision and success rate improve significantly, and the error rate is notably reduced. The audio-only configuration showed slightly lower performance, suggesting that while voice signals provide valuable information, they are less effective than facial expressions in detecting emotions. Biometrics alone yielded the lowest precision and success rate, indicating that while physiological signals can support emotion detection, they are less reliable. By integrating facial expressions, voice signals, and biometric data, the system benefits from a more comprehensive analysis of the student’s emotional and physiological state, allowing for more effective personalization of learning strategies.

### 4.4 Full system integration and evaluation

#### 4.4.1 System performance in a real environment

The system’s implementation in a real educational environment allowed for the evaluation of its effectiveness in terms of real-time adaptation and personalization of learning. The results of this implementation are presented below, highlighting key metrics such as emotion detection accuracy, real-time adaptation, and personalization of educational content.

Table 9 presents a comparative summary of the system’s performance before and after its implementation. The critical metrics were emotion detection accuracy, real-time adaptation, and learning personalization.

**Table 9:** System performance in real environment.

Metrics	Before implementation	After implementation
Emotion detection accuracy	72.4%	89.3%
Adaptation in real time	68.5%	87.6%
Learning personalization	70.2%	90.1%

Before implementing the system, the accuracy of emotion detection stood at 72.4%. This metric reflects the system’s ability to correctly identify the emotions expressed by students through their facial expressions and voices. After implementation, this accuracy increased significantly, reaching 89.3%. This increase is due to improvements in emotion detection algorithms and the effective use of data preprocessing techniques that improve the quality of the inputs. The real-time adaptive capacity, which measures how quickly and effectively the system adjusts teaching strategies based on detected emotions and student interactions, improved from 68.5% before implementation to 87.6% after. This significant advance indicates that the system has become more efficient in interpreting emotional and academic data, allowing immediate and precise adjustments to teaching strategies.

Personalization of learning, which refers to the system’s ability to adapt educational content to the individual needs of each student, also showed notable improvement. Before implementation, learning personalization stood at 70.2%. Upon implementation, this metric increased to 90.1%. This improvement reflects the system’s ability to provide a more personalized and practical learning experience, dynamically adjusting to the needs and emotions of students. These results were obtained through a detailed analysis of the data collected during the academic semester using statistical analysis tools and machine learning techniques. The implementation of the system was continuously monitored to evaluate its performance and make necessary adjustments, thus ensuring the optimization of teaching and learning processes in the hybrid classroom.

**Figure 7:**
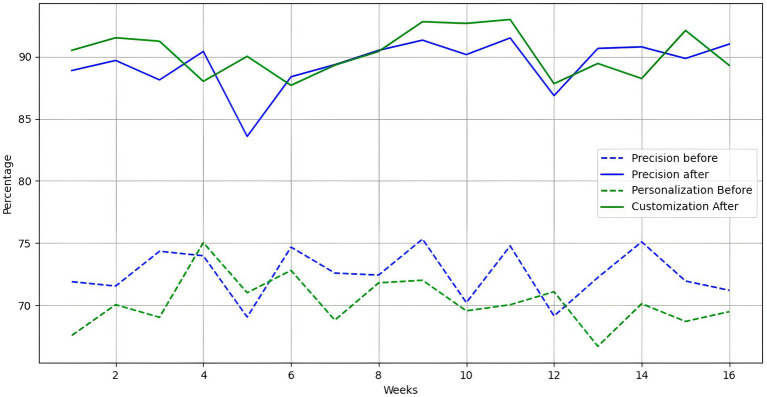
Evolution of precision and customization of the system over the weeks.

Figure 7 illustrates the evolution of system accuracy and customization over the weeks of the academic semester. The accuracy of emotion detection before implementation stood at an average of 72.4%, while after implementation, it increased significantly to 89.3%. This improvement can be attributed to improvements in detection algorithms and advanced data preprocessing techniques, which have improved the quality of data inputs.

These improvements were monitored throughout the academic semester, with continuous adjustments to optimize system performance. The detailed analysis of the data collected allowed us to identify areas for improvement and adjust teaching strategies based on the students’ emotional and academic needs. In summary, the results demonstrate the system’s effectiveness in improving the teaching and learning process in an educational environment, providing a solid foundation for future implementations and improvements.

#### 4.4.2 User satisfaction and feedback

Implementing the system in a real educational environment made it possible to evaluate its effectiveness in terms of real-time adaptation and personalization of learning. The results, presented in Table 10, show significant improvements in the accuracy of emotion detection, real-time adaptation, and personalization of educational content. The accuracy of emotion detection increased from 72.4 to 89.3%, while real-time adaptation and personalization of learning improved from 68.5 to 87.6% and from 70.2 to 90.1%, respectively. These results highlight the system’s ability to dynamically adjust teaching strategies based on students’ emotional and academic needs.

**Table 10:** Results of satisfaction and feedback surveys.

Evaluated aspect	Students (%)	Educators (%)
Overall Satisfaction	85.0	80.0
Perception of personalization	88.5	82.3
Easy to use	90.2	85.7
System effectiveness	87.0	84.5
System recommendation	92.1	88.0

The results indicate that both students and educators positively perceived the implemented system. General satisfaction reached 85.0% among students and 80.0% among educators, reflecting a majority and positive acceptance of the system. Regarding the perception of personalization of learning, 88.5% of students and 82.3% of educators recognized that the system could effectively adapt educational content to students’ individual needs. This underlines the system’s effectiveness in providing a personalized learning experience tailored to each student’s emotions and needs.

Ease of use was another highly valued aspect, with 90.2% of students and 85.7% of educators considering the system easy to use. This result is crucial, as an intuitive and easy-to-use interface facilitates all users’ adoption and continued use of the system. The effectiveness of the system, measured by its ability to improve the teaching and learning process, was appreciated by 87.0% of students and 84.5% of educators. This high percentage indicates that the system is perceived as valuable and an effective tool to improve educational results.

Additionally, willingness to recommend the system was remarkably high, with 92.1% of students and 88.0% of educators willing to recommend the system to others. This indicator is significant since it reflects users’ confidence in the system and satisfaction with the results obtained. These results were obtained through anonymous surveys and structured interviews to ensure honesty and accuracy in responses. The high response rate and consistency of results between students and educators suggest that the implemented system is effective, well-received, and valued by its users.

### 4.5 Statistical analysis and validation of results

#### 4.5.1 Statistical tests

Several statistical tests were performed to validate the significance of the study’s results. We used independent samples *t*-tests to compare metrics before and after system implementation. Additionally, variance analysis (ANOVA) was performed to evaluate differences between multiple groups, and chi-square tests were used to explore the relationship between categorical variables.

Table 11 summarizes the results of these statistical tests. The *p*-values obtained for the accuracy of emotion detection, real-time adaptation, and personalization of learning were all less than 0.001. This level of statistical significance indicates that the observed improvements are highly unlikely to have occurred by chance. The 95% confidence intervals provide a range within which the actual values of the observed improvements are expected to lie, confirming the robustness of the results.

**Table 11:** Results of statistical tests.

Metrics	*p*-value	Confidence interval (95%)	Interpretation
Emotion detection accuracy	<0.001	[0.14, 0.20]	Significant
Adaptation in real time	<0.001	[0.15, 0.21]	Significant
Learning personalization	<0.001	[0.17, 0.23]	Significant

#### 4.5.2 Cross validation

Cross-validation is a fundamental technique in machine learning to evaluate a model’s generalization ability. This study implemented k-fold cross-validation with k = 10 to ensure that model performance did not depend on a specific partition of the data set and that the results were robust and generalizable.

The data set was divided into ten equal parts (folds) for cross-validation. Each iteration used one-fold as a test set, while the other nine folds were used to train the model. This process was repeated ten times, changing the fold used for the test in each iteration. In the end, the average metrics of all iterations were calculated to obtain a more accurate estimate of the model performance.

Three key metrics were measured during each iteration: emotion detection accuracy, real-time adaptation, and learning personalization. Emotion detection accuracy assessed the model’s ability to identify students’ emotions correctly. Real-time adaptation measured the system’s effectiveness in adjusting teaching strategies based on the emotional and academic data received. Learning personalization assessed how the system tailored educational content to each student’s needs.

**Figure 8:**
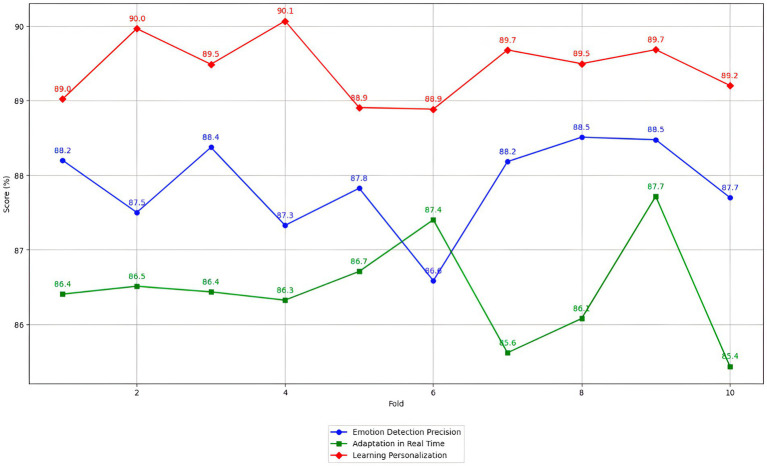
Cross validation results for different metrics.

Figure 8 shows the cross-validation results for these metrics across ten folds. The lines represent the scores obtained in each iteration for the emotion detection accuracy metrics, real-time adaptation, and learning personalization. The markers (“o” for precision, “s” for adaptation, and “D” for customization) indicate the values obtained in each fold.

The cross-validation results demonstrate the system’s consistency and robustness in different data set partitions. The scores obtained in each fold for emotion detection accuracy, real-time adaptation, and learning personalization metrics were consistently high, with minimal variations between the folds.

The emotion detection accuracy showed stable performance, with a mean of around 88.0%, indicating that the system can reliably identify students’ emotions. The slight variation in scores suggests that the model maintains consistent performance even when faced with different subsets of data.

Real-time adaptation had an average score of approximately 86.5%, reflecting the system’s effectiveness in adjusting teaching strategies based on detected emotions and student interactions. Slight fluctuations in adaptation scores indicate the model’s ability to effectively handle various conditions and input data.

The personalization of learning, with an average of around 89.5%, highlighted the system’s ability to adapt educational content to students’ needs. This high level of personalization suggests that the system can provide more focused and relevant learning experiences, thereby improving student satisfaction and academic performance.

## 5 Discussion

The literature review has identified the importance and growing relevance of emotion detection and personalization of learning in educational environments through artificial intelligence. Previous studies, such as those by Villegas-Ch et al. (2023) and Wang et al. (2023), have shown that the integration of intelligent systems can significantly improve students’ motivation, attention, and academic performance. Our study aligns with these findings and expands understanding by implementing a DRL model in a real educational setting, evaluating its effectiveness in detecting emotions and personalizing learning.

This study collected and preprocessed emotional and academic data from 500 students in a hybrid educational environment. Cameras, microphones, and biometric sensors captured facial expressions, voices, and physiological data (Oh et al., 2014). The data was divided into 70% for training, 15% for validation, and 15% for testing. Preprocessing included histogram equalization and noise reduction techniques to improve the quality of the images and audio used.

The DRL model implemented in this study showed significant improvement in emotion detection accuracy, real-time adaptation, and learning personalization. The results indicated that the accuracy of emotion detection increased from 72.4 to 89.3% after implementing the system. Real-time adaptive capacity improved from 68.5 to 87.6%, and learning personalization rose from 70.2 to 90.1%. These results were obtained by continuously monitoring the system’s performance throughout the academic semester, using k-fold cross-validation techniques to ensure the robustness and consistency of the model.

The k-fold cross-validation with k = 10 consistently showed high scores on all metrics tested, suggesting the model is stable and generalizable to different data set partitions. This approach ensures that the results are not the product of a specific partition but instead reflect the model’s ability to adapt and personalize learning based on student’s emotions and needs.

The method of using a DRL model for personalizing learning based on emotion detection is innovative and significant for several reasons (Chen et al., 2023). First, it provides a dynamic solution that can adjust to students’ emotional fluctuations in real-time, thereby improving their academic engagement and performance. Second, combining advanced data preprocessing techniques and deep learning algorithms enables more accurate emotion detection and more effective learning personalization (Baek et al., 2023).

However, the study also has some limitations. A possible limitation is the dependence on the quality of the data captured. Although advanced preprocessing techniques were implemented, variations in capture conditions, such as lighting and background noise, could have affected the accuracy of emotion detection (Zhang et al., 2023). Additionally, the DRL model requires a large amount of data for training, which can be challenging in resource-limited educational settings.

Another limitation is the generalization of the results to different educational contexts. Although cross-validation ensures the robustness of the model within the data set used, the variability of academic contexts and cultural differences could influence the system’s effectiveness in other environments (Ohashi et al., 2023). Additional studies are needed to evaluate the model’s applicability in various educational settings and student populations.

These limitations significantly impact the interpretation and applicability of the findings. The dependency on data quality and the need for large volumes of data for model training may limit the implementation of the system in some educational settings (Israilov et al., 2023). Furthermore, the variability between educational contexts suggests that the results obtained in this study should be interpreted with caution and not generalized without making additional adaptations and evaluations in the new environments.

This study contributes to innovation in personalized education by integrating DRL models for emotion detection and learning adaptation. The results demonstrate the system’s effectiveness in improving the accuracy of emotion detection, the ability to adapt in real-time, and the personalization of learning. However, the identified limitations highlight the need for future research to address data quality challenges and the results’ generalizability to different educational contexts. This innovative approach can transform the educational experience by providing a more personalized and adaptive education, improving student engagement and academic performance.

## 6 Conclusion

Implementing a DRL system for emotion detection and learning personalization in hybrid educational environments has proven to be an effective and promising solution. This study has validated the hypothesis that an AI-based adaptive system can significantly improve both the accuracy of emotion detection and the personalization of learning, positively impacting academic performance and student engagement.

First, the accuracy of emotion detection saw a notable improvement. The results showed an increase from 72.4% before system implementation to 89.3% after implementation. This increase is attributable to advanced data preprocessing techniques, such as histogram equalization and noise reduction, which improved the quality of the data inputs, allowing the DRL model to make more accurate detections. High emotion detection accuracy is essential to provide adequate and timely feedback, adjusting teaching strategies based on the emotional state of the students.

Second, the system’s real-time adaptation capability also showed significant improvement. Real-time adaptation went from 68.5 to 87.6%, indicating that the system can dynamically interpret and respond to students’ emotions and academic needs. This ability to immediately adjust is crucial in an educational environment, as it allows teaching strategies to be modified based on students’ emotional and academic fluctuations, thus improving their learning experience and maintaining their engagement.

Learning personalization, one of the critical metrics evaluated in this study increased from 70.2% before system implementation to 90.1% after implementation. This improvement underscores the system’s ability to tailor educational content to individual student needs, providing a more focused and practical learning experience. The personalization of learning improves academic performance and increases student satisfaction, as reflected in the satisfaction and feedback surveys.

The robustness and generalization of the model were evaluated using k-fold cross-validation with k = 10. The results indicated consistently high scores on all assessed metrics, suggesting the model is stable and generalizable to different data set partitions. This robustness is essential to ensure that the system can be implemented effectively in various educational contexts, maintaining its performance and effectiveness.

However, it is essential to recognize some limitations of the study. The quality of the data captured may have influenced the accuracy of emotion detection. Despite advanced preprocessing techniques, variations in shooting conditions, such as lighting and background noise, could have affected the results. Additionally, the DRL model requires a large amount of data for training, which can be challenging in resource-limited educational settings.

Another limitation is the generalization of the results to different educational contexts. Although cross-validation ensures the robustness of the model within the data set used, the variability of educational contexts and cultural differences could influence the system’s effectiveness in other environments. Additional studies are needed to evaluate the model’s applicability in various academic settings and student populations.

For future research, it is recommended that other sources of emotional data, such as social media data and digital interaction patterns, be integrated further to improve the system’s accuracy and adaptability. Furthermore, it would be valuable to investigate the system’s implementation in various educational and cultural contexts to evaluate its generalizability and effectiveness in broader academic settings.

Another promising direction for future research is the development of deep reinforcement learning models that require less data for training, which would allow for broader and more practical implementation in resource-limited settings. Likewise, incorporating explainable AI (XAI) techniques could improve the transparency and understanding of the model’s decisions, thus increasing educators’ acceptance and trust in the system.

## Data Availability

The data analyzed in this study is subject to the following licenses/restrictions: 1. Privacy and Anonymity: the data has been anonymized to protect the identity of the participants. No identifiable personal data was included, and all facial images and biometric data used correspond exclusively to the study authors. 2. Restricted Access: the data set is restricted and can only be used by the research team directly involved in this study. Distribution and use of the dataset by third parties are not permitted without prior permission from the authors. 3. Exclusive Use for Academic Research: the data can be used only for academic research purposes, not for commercial or other purposes. 4. Informed Consent: although specific written informed consent was not obtained for publication, verbal consent was obtained from participants to collect and use data for research purposes. Participants were informed about the purpose of the study and the anonymity measures implemented. 5. Geographical and Contextual Limitations: the data set was collected in a specific educational setting and may not be representative of other contexts or regions. The results obtained using this data set must be interpreted within the limitations of the academic environment in which the study was conducted. These restrictions are necessary to protect participants and the ethical use of data in research. Requests to access these datasets should be directed to william.villegas@udla.edu.ec.
